# Specificity and biologic activities of novel anti-membrane IgM antibodies

**DOI:** 10.18632/oncotarget.12506

**Published:** 2016-10-09

**Authors:** Rachel S. Welt, Jonathan A. Welt, David Kostyal, Yamuna D Gangadharan, Virginia Raymond, Sydney Welt

**Affiliations:** ^1^ American Museum of Natural History, New York, NY, USA; ^2^ Welt Bio-Molecular Pharmaceutical, LLC., Armonk, NY, USA; ^3^ ARDL, Inc., Akron, OH, USA; ^4^ Biogent, LLC., Armonk, NY, USA

**Keywords:** monoclonal antibody, membrane IgM, B-cell receptor, leukemia, lymphoma

## Abstract

The concept that the B-cell Receptor (BCR) initiates a driver pathway in lymphoma-leukemia has been clinically validated. Previously described unique BCR Ig-class-specific sequences (proximal domains (PDs)), are not expressed in serum Ig (sIg). As a consequence of sequence and structural differences in the membrane IgM (mIgM) μ-Constant Domain 4, additional epitopes distinguish mIgM from sIgM. mAbs generated to linear and conformational epitopes, restricted to mIgM and not reacting with sIgM, were generated despite the relative hydrophobicity of the PDm sequence. Anti-PD mAbs (mAb1, mAb2, and mAb3) internalize mIgM. Anti-mIgM mAb4, which recognizes a distinct non-ligand binding site epitope, mediates mIgM internalization, and in low-density cultures, growth inhibition, anti-clonogenic activity, and apoptosis. We show that mAb-mediated mIgM internalization generally does not interrupt BCR-directed cell growth, however, mAb4 binding to a non-ligand binding site in the mIgM PDm-μC4 domain induces both mIgM internalization and anti-tumor effects. BCR micro-clustering in many B-cell leukemia and lymphoma lines is demonstrated by SEM micrographs using these new mAb reagents. mAb4 is a clinical candidate as a mediator of inhibition of the BCR signaling pathway. As these agents do not bind to non-mIgM B-cells, nor cross-react to non-lymphatic tissues, they may spare B-cell/normal tissue destruction as mAb-drug conjugates.

## INTRODUCTION

The B-cell Receptor Complex (BCRC) emerged as a molecular target for the therapy of leukemia and lymphoma with the advent of personalized anti-idiotype (anti-id) monoclonal antibody (mAb) technology [1, 2]. Complementarity determining regions (CDRs) of cell surface membrane-Immumoglobulins (mIg) were recognized as both patient-unique “tumor specific” targets and as the critical regulatory element in the neoplastic process [3–8]. A “proof-of-principle” clinical study published in 1998 demonstrated that patient-specific, custom made anti-id mAbs, given alone or with other agents, induced objective measurable and durable responses [8]. Of note in this report was that patients in prolonged clinical remission harbored “dormant tumor cells” detectable by PCR [8]. These findings suggest that anti-id therapy can induce lineage-specific cell-differentiation and/or pathway regulatory effects [8].

An investigation of the mechanisms of anti-id resistance demonstrated that CDR sequence mutation variants, rather than loss of the mIg structure, were responsible for treatment failure [9–11]. Thus, signaling emanating from the BCRC with new mutated CDRs was responsible for persistence of the malignant phenotype. Subsequently, molecular studies demonstrated that Chronic Lymphocytic Leukemia (CLL) B-cells were in a constitutively activated state, recapitulating normal receptor-antigen-mediated activation, leading to proliferation and differentiation programs normally initiated by the BCRC [12, 13]. The complexity of these initiating activation steps is further emphasized by the observation that BCRC signaling can also be propagated through non-Immune-receptor Tyrosine-based Activation Motifs (ITAM) or by endocytosed receptors [14–15]. These findings support the contention that anti-id mAbs modulate tonic BCRC-mediated B-cell signaling leading to differentiation of malignant cells, dormancy, and/or induction of apoptosis [3–8]. Based on these observations, key downstream pathways of the BCRC have been identified and small molecule tyrosine kinase inhibitors (TKI) have been developed. The robust anti-tumor effects of these agents have now been demonstrated in clinical trials and provide “proof-of-principle” in support of the hypotheses regarding the central role of BCRC signaling in the molecular biology of B-cell malignancies [16–20]. Therefore, the development of additional agents to control the constitutively activated BCRC signaling is warranted, especially those targeting the BCR itself.

As a consequence of mIgM sequence homology to serum IgM (sIgM), specific B-cell mIgM targeting *in vivo* was thought not to be feasible, except for the anti-id, patient-specific CDR approach. However, the subsequent finding of unique class-specific sequences identified in mIg receptors, designated as “proximal domains” (PDs), that are not contained in the corresponding secreted Ig protein sequences (mRNA splice variants) (Genbank), opened new discovery pathways. These PD sequences represent potential cell surface epitope targets specific to each Ig class. mAbs reacting with the mIgE PD have been demonstrated to induce apoptosis [21]. Thus, the PD may be critical in transmitting mIg receptor transmembrane-signaling to the closely associated CD79α/β cytoplasmic tyrosine kinase (TK), and anti-PD mAbs may, in general, be able to modulate signaling [21]. This concept that mAbs that do not bind to, or block receptor ligand-binding sites, their ligands, or receptor dimerization sites, but can be potent inhibitors of receptor TK-mediated signaling, has already been established *in vitro* [22] and validated in the clinic.

Major alterations in the PD-μConstant Domain 4 (μC4) juncture further differentiate sIgM and mIgM and provide additional neo-epitopes and functional capabilities for specific targeting. For example, the μC4 domain of mIgM is differentiated from sIgM μC4 by a 20 amino acid truncation, loss of the J-chain binding site, and loss of a glycosylation site, which taken together generate unique epitopes associated with a new functional site: an active mIgM clustering/signaling domain [23–27].

Here we present the biologic effects of novel anti-PD mAbs. In contrast to the apoptotic effects observed in the anti-mIgE-PD system, only one of the anti-PDm mAbs significantly inhibited cell growth or induced apoptosis [28]. This mAb, with partial conformation-dependent binding spanning the PDm-μC4 juncture, manifests receptor internalization, cell growth inhibition, anti-clonogenic activity [29], anti-stem-cell activity [30], and apoptosis in low-density cultures [31].

## RESULTS

### Generation of hybridoma clones

Because the mIgM PD peptide is relatively hydrophobic, generating high avidity mAbs required novel immunization strategies. Its 13-mer sequence is comprised of five hydrophobic amino acids V, A, F, and two Gs, in addition to amino acid S which has a relatively low hydrophobicity index. Thus, stabilizing these peptides with carrier immunogens was essential for immunization and *in vitro* screening assays. Given the hydrophobicity of the PDm sequence, it was initially unclear whether it was partially contained in the plasma membrane or was completely in the extracellular space and accessible for mAb binding.

With the goal of specifically modulating mIgM-CD79α/β signaling, mAbs targeting the PDm sequence and the contiguous proximal extra-cellular domain of the mIgM (μC4) were generated. Proprietary immunization techniques for hydrophobic peptide immunogens were employed. Panels of peptide-specific mAbs detecting the 13-mer peptide PDm sequence (EGEVSADEEGFEN), specific for mIgM, and the 18-mer peptide PDg sequence (ELQLEESCAEAQDGELDG), specific for mIgG, were generated first. Three candidate mAbs (mAb1, mAb2, and mAb3), detecting PDm, were selected for further testing. In these studies an anti-PDg mAb11.1 (mouse IgG1) served as both positive and negative isotype control mAb in specificity and biologic studies. The initial screening and clone selection which yielded mAb1, mAb2, and mAb3 was based on ELISA, Hemagglutination (HA), Western blots, and Scanning Immune Electron Microscopy (SEM) assays, all of which demonstrated binding to (1) PDm peptide, (2) mIgM cell lysate protein fractions, and (3) cultured mIgM+ expressing cell lines: CA 46 (CRL 1648), SU-DHL-5 (CRL 2958), Ramos (CRL 1596), Namalwa (CRL 1432), ST 486 (CRL 1647), MC 116 (CRL 1649), and HT (CRL 2260).

Using a high affinity anti-PDm mAb (mAb1), NP-40 cell lysates containing mIgM were immune-affinity chromatography purified and used to immunize additional sets of mice. From these immunizations, second-generation mAbs detecting conformational BCRC epitopes, but not reacting with sIgM in ELISA assays and Western blots, were collected. One of these mAbs, designated mAb4, is differentiated by manifesting additional biologic activities, such as inducing B-cell growth inhibition, as assessed by MTT technology applied to clonogenic limiting dilution assays [22]. This finding emphasizes the need to probe native proteins as a source of biologically active mAbs. These four mAbs, designated mAb1, mAb2, mAb3, and mAb4 (collectively referred to as the “mAb panel”) were selected for further studies.

### Specificity analyses

Serial assays testing each hybridoma clone's specificity were used to select and further characterize the clinical candidates comprising the final mAb panel. In all specificity assays (Table 1A–1E), isotype-matched IgG1 and IgG2 antibodies (Abs) served as negative controls and anti-mIgG and anti-huIgM served as positive controls for the specified substrate (four columns on the right hand side of the tables). The first set of screens employed plates coated with molecular constructs consisting of (1) carrier alone, (2) peptides alone, (3) carrier with C- or N-terminal conjugation and peptides, (4) purified membrane fractions, and (5) two isomers of the PDm (Table 1A). As expected those constructs that lacked the PDm sequence were negative (Table 1A: Rows 1, 3, 5, 6, 8, 10) while those that contained PDm were positive (Table 1A: Rows 2, 4, 7, 9, 11, 12). Table 1A (Rows 13 and 14) shows PDm peptides with a V to N amino acid substitution at position 4 (Isomer 1) and an A to E substitution at position 6 (Isomer 2). mAb4 was the only one of the four mAbs to be significantly affected by these changes suggesting that it is sterically inhibited.

**Table 1A:** Specificity Analysis of mAbs: ELISA assay of immunogen reactivity

		Antibody Probe							
	Molecular Constructs-Immunogens	mAb1	mAb2	mAb3	mAb4	IgG1 isotype control	IgG2 isotype control	Anti-mIgG mAb 11	Anti-hu-IgM
**1**	KLH	0.1	0.1	0.1	0.1	0.1	0.1	0.1	0.1
**2**	**KLH-PDm**	**4.0**	**4.0**	**4.0**	**0.5**	0.1	0.1	0.1	0.1
**3**	KLH-PDg	0.1	0.1	0.1	0.1	0.1	0.1	**3.8**	0.2
**4**	**PDm-KLH**	**4.0**	**4.0**	**4.0**	**0.9**	0.1	0.1	0.1	0.1
**5**	PDg-KLH	0.1	0.1	0.1	0.1	0.1	0.1	**3.2**	0.1
**6**	MAP	0.2	0.2	0.2	0.1	0.1	0.1	0.1	0.1
**7**	**MAP-PDm**	**4.0**	**4.0**	**4.0**	**0.8**	0.1	0.1	0.1	0.1
**8**	MAP-PDg	0.2	0.1	0.1	0.1	0.1	0.1	**3.5**	0.2
**9**	**PDm**	**4.0**	**4.0**	**4.0**	**0.8**	0.1	0.1	0.1	0.1
**10**	PDg	0.2	0.1	0.1	0.1	0.1	0.1	**3.3**	0.1
**11**	**P-F mIgM**	**3.1**	**2.8**	**2.6**	**4.0**	0.3	0.1	0.1	**4.0**
**12**	**P-F + IA-mIgM**	**3.3**	**2.5**	**2.3**	**4.0**	0.1	0.2	0.1	**4.0**
**13**	**PDm Isomer 1**	**3.8**	**3.2**	**3.0**	**0.9**	0.1	0.1	0.1	0.1
**14**	**PDm Isomer 2**	**3.2**	**3.0**	**3.2**	**0.7**	0.1	0.1	0.1	0.2

P-F cell lysates prepared by Perfect FOCUS^TM^, IA - immune affinity chromatography

**Table 1B:** Specificity Analysis of mAbs: Probing of Lymphatic and Non-Lymphatic cell lines by HA/ELISA

	B-Cells	mAb1	mAb2	mAb3	mAb4	IgG1 isotype control	IgG2 isotype control	Anti-mIgG mAb11	Anti-hu-IgM
**1**	IgM k (4)	**+/+**	**+/+**	**+/+**	**+/+**	−/−	−/−	−/−	**+/+**
**2**	IgM l (3)	**+/+**	**+/+**	**+/+**	**+/+**	−/−	−/−	−/−	**+/+**
**3**	IgG k (1)	−/−	−/−	−/−	−/−	−/−	−/−	**+/+**	−/−
**4**	IgG l (1)	−/−	−/−	−/−	−/−	−/−	−/−	**+/+**	−/−
**5**	IgE (1)	−/−	−/−	−/−	−/−	−/−	−/−	−/−	−/−
	**Non-Lymphatic Cell Lines**	mAb1	mAb2	mAb3	mAb4	IgG1 isotype control	IgG2 isotype control	Anti-mIgG mAb11	Anti-gpA33, EpCa
**6**	Colon (12)	−/−	−/−	−/−	−/−	−/−	−/−	−/−	**+/+**
**7**	Breast (9)	−/−	−/−	−/−	−/−	−/−	−/−	−/−	**+/+**
**8**	Lung (9)	−/−	−/−	−/−	−/−	−/−	−/−	−/−	**+/+**
**9**	Melanoma (2)	−/−	−/−	−/−	−/−	−/−	−/−	−/−	−/−

The specific cell lines used are given in the Materials and Methods. Results indicate whether a particular assay gave a positive (+) or negative (−) result in a modified HA assay and ELISA assay. (n) sample size

**Table 1C:** Specificity Analysis of mAbs: inhibition of ELISA by molecular constructs

	Molecular Construct	mAb1	mAb2	mAb3	mAb4	IgG1 isotype control	IgG2 isotype control	Anti-mIgG mAb 11	Anti-hu-IgM
**1**	KLH	**3.9**	**3.5**	**3.6**	**3.8**	**0.1**	**0.1**	**0.1**	**3.9**
**2**	KLH-PDm	**0.7**	**0.3**	**0.4**	**3.8**	**0.1**	**0.1**	**0.2**	**3.9**
**3**	KLH-PDg	**4.0**	**3.6**	**3.7**	**4.0**	**0.1**	**0.1**	**0.1**	**3.8**
**4**	PDm	**0.4**	**0.5**	**0.4**	**3.8**	**0.1**	**0.1**	**0.2**	**3.9**
**5**	PDg	**4.0**	**3.8**	**3.2**	**3.9**	**0.1**	**0.1**	**0.3**	**3.7**
**6**	P-F mIgM	**0.2**	**0.3**	**0.3**	**0.2**	**0.1**	**0.1**	**0.2**	**0.2**
**7**	P-F + IA mIgM	**0.3**	**0.4**	**0.2**	**0.2**	**0.1**	**0.1**	**0.2**	**0.2**

P-F cell lysates prepared by Perfect FOCUS^TM^ technology, IA - immune affinity chromatography

**Table 1D:** Specificity Analysis of mAbs: ELISA assay of biological (serum) samples

	Biologic Specimens	mAb1	mAb2	mAb3	mAb4	IgG1 isotype control	IgG2 isotype control	Anti-mIgG mAb11	Anti-hu-IgM
**1**	Normal serum (6)	**0.2**	**0.2**	**0.3**	**0.3**	**0.2**	**0.3**	**0.4**	**4.0**
**2**	Purified IgM (1)	**0.3**	**0.4**	**0.3**	**0.3**	**0.3**	**0.2**	**0.2**	**4.0**
**3**	W-M serum (2)	**0.3**	**03**	**0.3**	**0.2**	**0.2**	**0.2**	**0.3**	**4.0**

(n) - sample size, W-M - Waldenstrom macroglobulinemia

**Table 1E:** Specificity Analysis of mAbs: inhibition of ELISA by biological constructs

	Biological Construct	mAb1	mAb2	mAb3	mAb4	IgG1 isotype control	IgG2 isotype control	Anti-mIgG mAb 11	Anti-hu-IgM
**1**	Normal serum (3)	**3.9**	**3.7**	**3.9**	**4.0**	**0.1**	**0.1**	**0.2**	**0.2**
**2**	Normal plasma (3)	**3.7**	**3.8**	**3.7**	**3.9**	**0.1**	**0.1**	**0.1**	**0.4**
**3**	W-M serum (2)	**3.8**	**3.8**	**3.8**	**4.0**	**0.1**	**0.2**	**0.1**	**0.1**
**4**	DLBCL serum (3)	**3.5**	**3.6**	**3.7**	**4.0**	**0.1**	**0.1**	**0.1**	**0.8**
**5**	NHL serum (3)	**3.4**	**3.3**	**3.7**	**3.9**	**0.1**	**0.1**	**0.2**	**0.7**
**6**	Breast Ca serum (3)	**3.6**	**3.7**	**3.6**	**4.0**	**0.1**	**0.1**	**0.2**	**0.3**
**7**	Colon Ca serum (3)	**3.8**	**3.4**	**3.8**	**4.0**	**0.1**	**0.1**	**0.1**	**0.3**
**8**	CLL serum (3)	**3.8**	**3.7**	**3.8**	**4.0**	**0.1**	**0.1**	**0.2**	**1.9**
**9**	CLL cells (3)	**0.2**	**0.2**	**0.1**	**0.1**	**0.1**	**0.1**	**0.2**	**0.6**

(n) sample size

**Figure 1:**
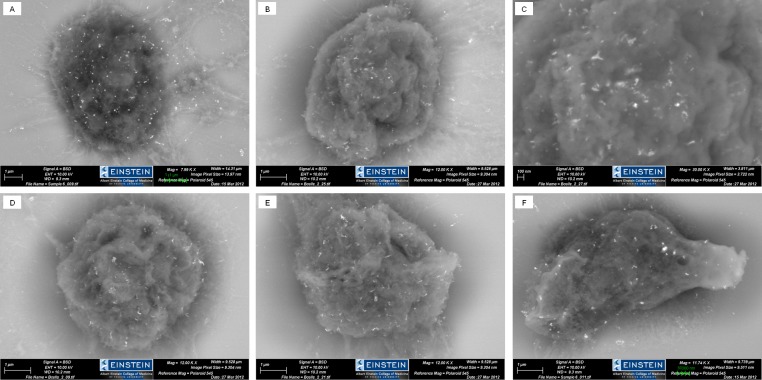

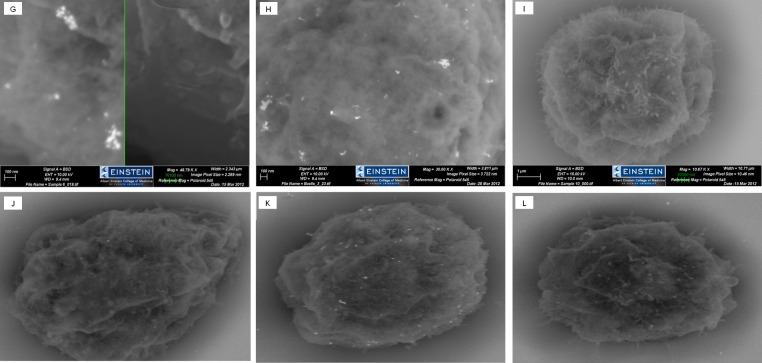
SEM of mAb binding to cell surfaces **A.**, **B.**, and **C.** show mAb4 binding to CRL 1648 cells. Anti-PD mAb1 **D.**, mAb2 **E.**, and mAb3 **F.** bind to CRL 1648. Isotype control mAb (IgG1) binding to CRL 1648 is shown in **J**. Various degrees of micro-clustering are identified in most cells examined, shown for mAb4 **G.** and mAb1 **H.** Polyclonal mouse anti-huIgM cell reactivity post mAb4-induced receptor internalization **I.**. Kinetics of mAb4 internalization at 5 **K.** or 30 mins **L.** post incubation, compared to glutaraldehyde-fixed cells exposed to mAb4 (A, B, and C) or control mAb **J.**

To determine whether the mAbs are capable of binding to the specific PDm sequence in the native state, and to eliminate the possibility of artifactual, non-specific, low-affinity hydrophobic binding, a modified HA assay of intact cells (see *Materials and Methods*) and ELISA assays using cell lysates were performed. The results are scored as either positive (+) or negative (−) and both lymphatic and non-lymphatic cells lines were tested in the HA and cell lysate assays. A portion of these cell lines were prescreened by RT-PCR to confirm mIgM expression. The results show that each of the mAbs recognizes cells or their lysates from only those cells containing the PDm, confirming their specificity for the native target (Table 1B: Rows 1 and 2). Negative control mIgG- and mIgE-expressing cells (Table 1B: Rows 3-5), as well as all of the Non-Lymphatic cell lines (Table 1B: Rows 6-9), were negative in both assays. We found that all cell lines expressing IgM by RT-PCR were also positive for mIgM by Western blot (data not shown). In some cell lines, mIgM expression was very low, < 10,000 receptors per cell as determined from SEM estimates (e.g. CRL 1648; Figure 1A-1C). Furthermore, reactivity with our panel of anti-PD mAbs suggested that when mIgM was detectable in cell lysates (ELISA assays), it was also present on the cell surface (HA assays) (Table 1B), correlating cell protein expression with cell surface expression.

Investigations with SEM also confirmed the cell-surface localization of the mIgM on intact viable target cells (Figure 1A-1C). Taken together, as cell lines were found to express mIgM in HA assays, cell lysate ELISA assays, SEM, and mIgM RT-PCR, these results suggest a strong correlation between mRNA expression, mIgM protein synthesis, and cell surface expression. These results are also in agreement with ATCC cell line mIgM expression data. In addition, HA/ELISA data indicated that the four mAbs do not cross-react nor detect any determinants on the surface of the non-mIgM expressing B-cell lines or the epithelial/non-lymphatic cell lines tested (*p* < 0.05) (Table 1B). Thus, these assays demonstrate both the restricted nature of mIgM expression, and lack of cross-reactive determinants in a variety of non-B-cell lineages. Therefore, the PDm sequence is not detected by immunologic means in non-mIgM-expressing cells. Isotype-matched control mouse mAbs, and anti-epithelial Abs were negative for B-cell lines, ruling out non-specific Fc receptor binding (Table 1B). Control positive and negative anti-mIgG, mAb11.1, and a control positive polyclonal goat anti-huIgM reagent demonstrated the specificity and robustness of the assay (*p* < 0.05) (Table 1B). Flow cytometry experiments detected mIgM as a “DIM” signal, due to low mIgM expression levels. Thus, this technology was considered inadequate for experiments examining mIgM cell surface expression and experiments measuring the reduction in signal due to antigen internalization (data not shown).

HA was originally designed as a methodology to rapidly assess Ab cell-surface binding to adherent target cells, and was adapted here for non-adherent or weakly-adherent cells (see *Materials and Methods: Hemadsorption/Hemagglutinin assays*). Using diluted test samples, this assay became especially sensitive in detecting non-rosetted cells. Thus, it was useful in confirming that virtually all cells within the positive cell lines were HA positive, and heterogeneity of mIgM expression, for positive and negative cells, was not detectable with any of the four mAbs tested and in any of the cell lines tested (Table 1B). The protein G-RBC also weakly detected IgG-expressing B-cells even without the addition of mAb, indicating its weak binding to surface-expressed mIgG. As this binding was blocked by pre-incubation of protein G-RBCs with control IgG2a, it indicates that the protein G-RBC HA reaction is mediated by the protein G-Ig binding. In these mIgG-expressing cells, HA background was as high as 30%.

ELISA assays testing the reactivity of the mAbs to the immunogen molecular constructs (bound to solid phase) further demonstrate the restrictive reactivity of the four mAbs in our panel. No reactivity was observed with carrier proteins keyhole limpet hemocyanin (KLH) or multiple antigen peptide (MAP), or with PDg when bound to KLH, MAP, or used without carrier. Observed reactivity was restricted to free or conjugated PDm (*p* < 0.05) (Table 1A).

A critical finding shown here is the relatively enhanced reactivity of mAb4 to purified native mIgM (Table 1A: Rows 11, 12) compared to its binding to PDm-containing structures (Table 1A: Rows 2, 4, 7, 9) particularly when compared to mAb1, mAb2, and mAb3 binding patterns. Both the Perfect FOCUS^TM^ lysate fraction, and the same fraction further enriched for mIgM by mAb1-Immune-affinity chromatography (Perfect FOCUS^TM^ + IA), showed preferred mAb4 binding (*p* < 0.05). These findings suggest that mAb4 has preferential binding to the intact native mIgM protein when compared to the PDm peptide and that its epitope may reside predominantly in the μC4 domain or may depend on this structure for proper conformation.

The two isomeric forms of the PDm (Table 1A: Rows 13, 14) appear to be detected by each of the mAbs with the same relative reactivity as the immunizing PDm or Perfect FOCUS^TM^ purified mIgM fraction (*p* < 0.05). These isomeric forms have a single amino acid variant each, substituting N for V and E for A at positions 4 and 6 in the 13-mer PDm, rendering these forms more hydrophilic than the most common isomer used in this study for immunization. These mAbs therefore cannot be used to distinguish the different mIgM isomeric forms and further investigations are needed to determine their biologic differences.

The positive control goat anti-huIgM reagent is not reactive with PDm or its conjugated structures, but does recognize the mIgM-purified samples (*p* < 0.05) (Tables 1A–1E). This indicates that common commercial human IgM preparations, used for immunizations to generate anti-huIgM research reagents, do not contain the PDm sequence or PDm is too weak in this context to induce an immune response. To further confirm the direct mAb binding ELISA assay (Table 1A), more sensitive inhibition of ELISA mAb binding assays are shown in Table 1C, confirming the highly selective specificity of these Abs.

To determine if, *in vivo,* mIgM-containing immunogenic PDm or μC4 are secreted or released from live cells by enzymatic means or by an apoptotic process, normal human serum, purified hu-IgM, and serum from Waldenstrom's Macroglobulinemia patients were each tested, bound to solid phase in ELISA assays, and found to be non-reactive with the mAb panel (*p* < 0.05) (Table 1D). To confirm these results, higher sensitivity inhibition assays were conducted using the same sera samples, now pre-incubated in antigen excess with the mAbs prior to use in ELISA assays, such as the direct binding to the Perfect-FOCUS^TM^ purified mIgM fraction (Table 1E). These inhibitory assays confirmed the results of the direct binding assays (*p* < 0.05). Following these initial experiments, an expanded panel of sera from normal patients, and a variety of patients with lymphatic and non-lymphatic malignancies, were tested, and demonstrated a lack of circulating mIgM in serum. In contrast, fresh frozen CLL cells (1 × 10^6^ cells in DMSO) almost completely absorbed each of the mAbs (1 μg/ml), and thus reduced mAb binding in direct binding assays to the Perfect-FOCUS^TM^ purified mIgM fraction (*p* < 0.05) (Table 1E). These fresh frozen CLL purified mononuclear cells separated by Ficoll-Hypaque technology were a source for NP-40 lysates and Perfect FOCUS^TM^ fractions for immune-affinity chromatography, immunizations, and ELISA assays.

SEM was used to investigate the cell binding patterns of the panel of mAbs to B-cell lines and to confirm the results of the HA and ELISA assays. The cell surface spatial array of mIgM receptor is poorly understood with regards to polymerization of mIgM by the μC4 clustering-domain, and to non-covalent CD79α/β complexing. We explored whether the PDm or μC4 clustering-domain is actively complexed on the cell surface and whether the mAb epitopes can still be detected in the presence of mIgM clustering. Except for PDm, class-specific PDs contain cysteines, which are hypothesized to be responsible for cross-linking mIg. mAbs detecting these PDs need to recognize the cross-linked PD. In the absence of cysteines in PDm, whether the hydrophobic nature of the PDm mediates its cross-linking is not supported by experimental data [25–27]. For this reason, SEM was carried out to provide additional evidence of mAbs binding to the cell surface of mIgM-expressing cells (Figure 1A-1F) but not to non-expressors. At higher magnification, “small” micro-clusters of mIgM were visualized by the gold-labeled goat anti-mouse Ig reagent (Figure 1G, 1H). As these “small” micro-clusters were visualized in glutaraldehyde-fixed cells, they are not a consequence of mAb-induced clustering, but rather represent *de novo* clustered mIgM. Similar complexes have been identified in diffuse large B-cell lymphoma cell lines (DLBCL); however, it is unclear whether they correspond to the micro-clustering previously described in DLBCL samples [25–27]. The two DLBCL cell lines both demonstrated large clusters likely representing the previously described micro-clustering [25–27]. The cell line CRL 1648 represents a B-cell line with relatively low mIgM expression based on “DIM” light chain reactivity by flow cytometric analysis, similar to CLL cells, and is shown in Figure 1A-1C. This cell line is not thought to be derived from a DLBCL patient. Similar “small' micro-clusters were found in all the B-cell lines examined regardless of their proposed original tissue diagnosis. These results demonstrate the accessibility of each mAb to their respective epitope in the mIgM clustered state.

### Fine molecular epitope mapping by competitive mAb binding and peptide inhibition

Epitope binding for each of the mAbs was determined using four complimentary assays, 1) mAb blocking of horseradish peroxidase (HRP)-labeled mAb binding to antigen in ELISA assays, 2) specific peptide inhibition of HRP-labeled mAb binding to antigen in ELISA assays, 3) specific peptide inhibition of mAb cell surface binding to target cells as determined by SEM, and 4) mAb cell surface binding to target cells as determined by SEM using isotype specific probes.

Protein labeling techniques for direct HRP conjugation to mAb allowed for testing of inhibition of mAb-HRP binding in solid-phase ELISA assays. By using excess unlabeled mAb to block labeled mAb-HRP binding (Table 2), the following groups of clones were defined: those blocking the labeled mAb (same epitope), those not blocking the labeled mAb (different epitope), and those partially blocking the labeled mAb (a near epitope or weaker binding). In separate experiments each mAb was labeled with HRP and the inhibitory unlabeled blocking mAb assay was repeated until all the clones were epitope defined. mAb1 did not block mAb2, mAb3, or mAb4, but is self-blocked by unlabeled mAb1 (*p* < 0.05); mAb2 did not block mAb1 or mAb4, but did partially block mAb3, and unlabeled mAb2 blocked itself (*p* < 0.05). These results were similar for mAb3 analysis showing partial blocking by excess mAb2 (*p* < 0.05). mAb4 demonstrated lack of blocking by mAb1, mAb2, and mAb3 but blocked itself (*p* < 0.05). The results suggest that for PDm, KLH-PDm, and Perfect FOCUS^TM^ + IA chromatography fraction, mAb1 and mAb4 each detected a distinct epitope while mAb2 and mAb3 detected another defined epitope that is partially shared. mAb4, while binding to PDm, showed relatively improved binding to purified mIgM (Perfect FOCUS^TM^ + IA) *vs*. PDm (*p* < 0.05), especially when compared to the patterns of reactivity of the other mAbs, again suggesting preferential binding to a naturally derived-PDm epitope or a conformational dependency provided by μC4.

**Table 2:** Molecular Epitope Mapping by Competitive mAb Binding

Blocked by mAb1	mAb1	mAb2	mAb3	mAb4	Control mAb
mIgM-PD	11	88	82	72	neg
mIgG-PD	neg	neg	neg	neg	neg
KLH-mIgM-PD	6	94	92	74	neg
mIgM-PD-KLH	10	92	91	52	neg
P-Focus + IA	13	90	89	96	neg
**Blocked by mAb 2**	**mAb1**	**mAb2**	**mAb3**	**mAb4**	**Control mAb**
mIgM-PD	80	2	23	69	neg
mIgG-PD	neg	neg	neg	neg	neg
KLH-mIgM-PD	67	4	24	68	neg
mIgM-PD-KLH	78	2	34	71	neg
P-Focus + IA	78	3	31	96	neg
**Blocked by mAb 3**	**mAb1**	**mAb2**	**mAb3**	**mAb4**	**Control mAb**
mIgM-PD	85	35	9	63	neg
mIgG-PD	neg	neg	neg	neg	neg
KLH-mIgM-PD	79	28	4	72	neg
mIgM-PD-KLH	87	36	9	62	neg
P-Focus +IA	82	39	10	85	neg
**Blocked by mAb 4**	**mAb1**	**mAb2**	**mAb3**	**mAb4**	**Control mAb**
mIgM-PD	93	87	84	12	neg
mIgG-PD	neg	neg	neg	neg	neg
KLH-mIgM-PD	82	88	95	14	neg
mIgM-PD-KLH	90	94	85	9	neg
P-Focus + IA	95	94	86	9	neg

Inhibition assays, testing 6-mer peptides for their ability to block the mAbs binding to peptide or the Perfect FOCUS^TM^ + IA purified fraction in solid-phase ELISA assays, were conducted to confirm the data from the mAb epitope mapping studies, and to more precisely define the target epitopes (Table 3). The 6-mer A-peptide (EGEVSA) and 6-mer B-peptide (EEGFEN) were used in molar excess (X100) compared to the mAb-HRP. These studies demonstrated that mAb4 detected a distinct epitope not substantially blocked by either the 6-mer A-peptide or B-peptide. While mAb1 was not blocked by the 6-mer B-peptide, mAb1 was strongly blocked by the 6-mer A-peptide (*p* < 0.05), and mAb2 and mAb3 were partially blocked by both 6-mer A-peptide and B-peptide (*p* < 0.05). It should be noted that the high level of hydrophobic amino acids in A-peptide: G, V, and A, and in B-peptide: G, and F; may affect results of this assay due to solubility characteristics. Thus, the partial blocking of mAb2 and mAb3 by the 6-mers may represent relative avidity or solubility issues. Taking these uncertainties together prevents clear epitope target designations for mAb2 and mAb3. mAb1 is blocked by the most hydrophobic sequences, emphasizing the unusual nature of this Ab. For this reason, we show the SEM demonstrating its cell binding in Figure 1D.

**Table 3:** Molecular Epitope Mapping by Competitive 6-mer Peptide Binding

Blocked by 6-mer A					
Solid Phase Target	mAb1	mAb2	mAb3	mAb4	Control mAb
mIgM-PD	28	45	61	81	neg
mIgG-PD	neg	neg	neg	neg	neg
P-Focus	19	53	54	90	neg
**Blocked by 6-mer B**					
**Solid Phase Target**	**mAb1**	**mAb2**	**mAb3**	**mAb4**	**Control mAb**
mIgM-PD	84	57	50	70	neg
mIgG-PD	neg	neg	neg	neg	neg
P-Focus	90	48	57	94	neg

In an effort to better assign a specific epitope target for these mAbs, additional studies were carried out using a 15-mer peptide, extending the 13-mer PDm into the μC4 domain. These studies indicated that mAb4 had a modest (<10%), but statistically insignificant increase in binding to the extended 15-mer compared to the 13-mer (p > 0.05), while there was no change in mAb2 and mAb3 binding. In addition, the 15-mer had low, non-significant blocking activity in mAb4 binding to the Perfect FOCUS^TM^ + IA purified fraction (data not shown). These data again suggest that mAb4 binds only weakly to the PDm, and more robustly to a proximal μC4 epitope not contained in the 15-mer, or that its epitope is partially conformational and it is not represented well in linear hydrophobic peptides, which may have alternative folding patterns.

SEM studies were used to confirm results of the ELISA blocking experiments. The binding of mAbs to glutaraldehye-fixed CRL 1648 cells was determined by SEM after incubation of the mAbs with excess PDm peptide or 6-mers (x500). mAb1, mAb2, and mAb3 binding to the cell surface of CRL 1648 was blocked by PDm peptide or their respective 6-mer, as seen by SEM testing, whereas mAb4 was not fully blocked, and binding to the CR-1648 cell surface, while reduced, was readily detected (data not shown).

To further examine the mAb4 epitope, SEM studies were carried out on fixed CRL 1648 cells incubated with mAb1. Isotype specific goat anti-mouse IgG1 gold-labeled reagent did not detect the bound IgG2b mAb1, but when the IgG1 mAb4 was subsequently added it was able to detect bound mAb4. The anti-mouse IgG1-gold reagent complex detected IgG1 mAb4, bound to the surface of glutaraldehyde-fixed CRL 1648 cells, pre-incubated with the IgG2b mAb2, indicating that mAb2 binds to a different epitope than mAb4 and does not block mAb4 cell binding (data not shown).

### mAb-induced receptor internalization

When viable cells were examined, initial SEM studies indicated that detection of cell surface bound mAb or mouse anti-huIgM sera was both time and temperature dependent. Incubation of mAb or polyclonal mouse anti-huIgM sera at 4°C for 30 mins was compared to incubation at 37°C for 30 mins. At 37°C, neither mAb nor mouse anti-huIgM reagent was detectable, but if incubated at 4°C, cell surface bound mAb and mouse anti-huIgM was detectable. These findings were interpreted as Ab modulation of receptor mIgM expression, being consistent with Ab-induced receptor internalization, a metabolic process that is reduced at 4°C. Fixation with glutaraldehyde was also demonstrated to stop this biologic internalization process and preserve detection of cell surface mIgM by bound Ab. Binding of these mAbs, as determined by SEM, to glutaraldehyde-fixed CRL 1648 B-cell line is shown in Figures 1A-1F and demonstrates the low density expression of these mIgM molecules.

Using protein G-RBCs for cell surface mAb detection, cells pre-fixed with glutaraldehyde prior to the addition of the mAbs or mouse anti-huIgM sera preserve cell surface mAb detection at both 37°C and 4°C (Table 4). These findings also indicate that the epitopes for the mAbs are preserved with glutaraldehyde fixation. SEM assays and protein G-RBC assays are both sensitive and robust, as in control experiments or isotype-matched HA, background gold-labeled goat anti-mouse Ig reagent (Figure 1I, 1J) or background rosetting, respectively, is not observed after glutaraldehyde fixation. A time-elapsed SEM series of micrographs is shown in Figure 1 (A and B, compared to K and L, respectively). At 15 mins (Figure 1K) and 30 mins (Figure 1L) of mAb4 incubation at 37°C, followed by fixation to stop the internalization process, the majority of mAb internalization has already occurred even at the earliest time points measured (Figure 1K). Background goat anti-mouse Ig labeled gold is not observed.

Additional SEM studies show that mAb4-mediated internalization of mIgM by incubation of CRL 1648 cells at 37°C for 30 mins resulted in a lack of detectable mIgM on the cell surface by testing cells with the mAb panel or polyclonal mouse anti-huIgM heavy chain reagent for binding after the mAb4 incubation (Figure 1L). For example, in an additional confirmatory experiment using protein G-RBC as the detection method, cells were exposed to mAb4 at 37°C for 30 mins, fixed, and then tested for mIgM expression using polyclonal mouse anti-huIgM heavy chain reagent (similar to Table 4, live cells at 37°C, 30 min). Lack of protein G-RBC rosetting indicates a lack of residual cell surface bound mAb4 and an absence of mouse anti-huIgM heavy chain reagent detection of residual cell surface mIgM. Similar experiments testing mAb1-, mAb2-, and mAb3-mediated internalization of mIgM by incubation of CRL 1648 cells at 37°C for 30 mins also resulted in a lack of detectable mIgM on the cell surface using mAbs or anti-huIgM heavy chain reagent (*p* < 0.05). These finding suggest that all mAbs in the panel mediate virtually complete mIgM internalization, and thus each mAb recognized all forms of mIgM as defined by the mouse anti-huIgM heavy chain reagent reactivity. Similar results were observed in all mIgM-expressing B-cell lines in our cell panel. At 1 μg/ml of mAb/10^6^ B-cells, internalization is complete at 30 mins (*p* < 0.05), suggesting this ratio represents a state of mAb excess for this cell line.

Table 4 (Rows 3 and 4) shows that, as with glutaraldehyde, low temperature also limited internalization. In addition, these results show that glutaraldehyde fixation did not substantially affect mAb4 binding to target (comparing results of Rows 1 and 2 to 3 and 4; Table 4). Timed experiments demonstrated that, by 30 mins at 37°C (Table 4: Rows 9, 10), the pre-treated cells' capacity to adsorb mAb4-HRP from solution is reduced, without a significant difference between PBS and acetate wash (pH 4.0) (*p* < 0.05), suggesting that mIgM and mAb4 are predominantly internalized by 30 mins and are no longer free on the cell membrane to bind mAb4-HRP after acetate washing. The lack of residual cell surface mIgM resulted in no reduction of mAb4-HRP binding to mIgM solid phase in ELISA assays because cell mIgM was already internalized by pre-incubation with mAb4 at 37°C. Thus, there was no mAb4-HRP absorption capacity by these cells whether they were washed with PBS or acetate (pH 4.0) (*p* < 0.05). The robust mAb-induced internalization is demonstrated by lack of cell surface mIgM available for inhibition of mAb4-HRP or anti-huIgM test reagents subsequently used in ELISA assays. Similar experiments with each mAb confirmed mIgM internalization.

Experiments demonstrate that increasing cell culture times with mAb4 (at 37°C) reduced the amount of cell surface mIgM as assessed by SEM, protein G-RBCs, and cell adsorption after the cells are washed in acid.

**Table 4:** Detection of mAb-Induced mIgM Internalization by Adsorption: binding of mAb4-HRP after adsorption with pre-treated cells washed with PBS or acetate

Cell treatment prior to exposure to mAb4	Cell treatment after exposure to mAb4, but prior to adsorption of mAb4-HRP	CRL 1648 % binding of adsorbed mAb4-HRP to P-F mIgM	CRL 1647 % binding of adsorbed mAb4-HRP to P-F IgM	CRL 1596 % binding of adsorbed mAb4-HRP to P-F IgM
Glutaraldehyde-Fixed	PBS	100	100	100
Glutaraldehyde-Fixed	Acetate	10	21	19
Live cells on Ice	PBS	92	95	88
Live cells on Ice	Acetate	12	18	20
Live, 37°C (5 min)	PBS	77	67	66
Live, 37°C (5 min)	Acetate	26	18	27
Live, 37°C (15 min)	PBS	78	81	72
Live, 37°C (15 min)	Acetate	48	44	56
Live, 37°C (30 min)	PBS	70	64	60
Live, 37°C (30 min)	Acetate	66	62	61

### Biologic activity

Due to the uniqueness of the amino acid sequence of PDm and low homology to other GenBank sequences, we hypothesized that PDm may be involved in propagating receptor signaling functional activity to the cytoplasmic CD79α/β TK signaling domain, as was reported for PDe [21]. Thus, growth, cell proliferation, apoptosis, and clonogenicity assays of CRL 1648 cells were performed to determine whether the anti-PDm mAbs modulated these processes.

### Limiting dilution assays and cell density experiments

**Figure 2:**
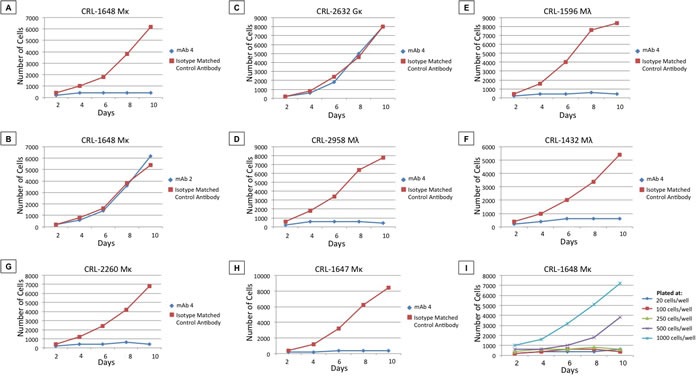
mAb4 inhibition of B-cell line growth **A.**-**H.** Blue curves represent number of cells of a cell line, specified in chart titles, cultured in the presences of 1 μg/ml of mAb4 or mAb2, as indicated, from two to ten days. Red curves represent the number of cells of these cell lines cultured in the presence of isotype-matched control IgG1. **I.** Number of viable cells (estimated from MTT optical density (OD)) across two to ten days in limiting dilutions assays with between 20 and 1,000 cells plated per well.

Limiting dilution assays of CRL 1648 cells, growing with or without 1 μg/ml of mAb4, demonstrated significant growth inhibitory effects and decrease in cell survival in 10-day culture experiments (Table 5, Figure 2). Marked inhibition of growth was observed in wells plated with 20-1,000 cells per well (20 *vs*. 500 *vs*. 1,000 cells/well). mAb4-treated wells did not achieve positive MTT signals, even when pooled, and remained at background level for the 10-day observation period. mAb4 depletion from solution by adsorption with fresh human CLLs resulted in a lack of detectable binding to PDm, the Perfect FOCUS^TM^ + IA purified fraction in solid-phase ELISA assays and growth inhibition properties. As cell growth in control wells achieved minimum cell numbers for positive MTT readings on days 2-4 of cell culture, the Student's t-test was only applicable to time points from days 2 to day 10, depending on the cell numbers plated and cell line. Growth curves shown in (Figure 2A, 2D-2I) present significantly different growth patterns in comparisons of mAb4 *vs*. isotype control mAb or comparisons of 1,000 *vs*. 500 *vs*. 250 or fewer cells/well.

Evidence of a significant caspase-dependent apoptotic effect by ELISA was found in residual cells at day 10 when compared to isotype control mAb (*p* < 0.05). At earlier time points no statistical caspase signal was detected (days 4-6). Thus, the major mechanism responsible for differential viable cell numbers, observed in days 4-10, was inhibition of single cell clone outgrowth as observed by phase contrast microscopy, rather than cell lysis. Caspase-positive wells were only obtained at days 8-10 (*p* < 0.05) indicating that apoptosis was a late event. The ability of stem cells to establish viable clones in limiting dilution conditions was impaired by mAb4 (Figure 2A), while mAb1, mAb2, and mAb3 only induced minor morphological changes and no change in cell numbers (Figure 2B, demonstrating lack of effect with mAb1 and mAb3). Changing growth conditions, such as % Fetal Calf Serum (FCS), or the use of normal human serum or CLL patient serum added to the complete media, did not affect mAb4 inhibitory activity (*p* < 0.05) (data not shown). Removal of mAb4 at ten days, re-culturing cells, and re-examining viability and clonability-efficiency of residual cells revealed no growth of clones, even in the absence of mAb4 (*p* < 0.05) (data not shown). Thus growth of cells after 20 days, ten days with mAb4 followed by ten without mAb4, yielded no microscopically visible viable cells or MTT signal.

Because these anti-tumor effects of mAb4 were observed in experiments testing limiting-dilution clonal growth conditions, we investigated whether the mechanism of mAb4 inhibition is consistent with the blocking of a soluble autocrine or paracrine growth factor, or is directly dependent on stem cell frequency. These hypotheses may be distinguished by dilutions of sets of identical numbers of plated cells in different media volumes. If the absolute number of stem cells determines the cell growth kinetics in the presence of mAb4, then stem cell number is critical regardless of volume. If, however, increasing the volume of media of these same sets of plated numbers of viable cells reduces cell growth kinetics, then a soluble factor concentration is the critical determinant of growth in the presence of mAb4 (Table 5). We therefore examined whether the number of cells plated per well, or relative cell density or cells/volume media, as determined by well size and media volume, correlate to optimal mAb4 anti-tumor effects. These experiments tested whether the mAb4 effect is directly modulating the functional activity of stem cells by inducing stem cell apoptosis or is competing with pro-survival and growth factors secreted by stem cells.

We found that mAb4 can suppress outgrowth of the most potent clonogenic cells, those capable of establishing single cell clones (at 1-5 cells per well). Other stem cells present at lower frequency can rescue clone development if sufficient cells are plated in the presence of mAb4. For example, if the critical stem cell was present at a frequency of 1 in 500 cells, this absolute number of 500 cells per well would be predicted to produce viable clones regardless of cell density/volume. However, we found a consistent inverse cell/volume-dependent growth pattern observed when identical numbers of cells were plated in increasing media volumes and surface areas available for growth in wells (Table 5). These findings suggest that cell density/volume media is also a determinant of B-cell survival in the presence of mAb4, along with absolute cell number, and thus concentrations of an as yet undefined secreted growth factor may be critical.

In this cell density/volume media experiment (Table 5), we observed significant differences (*p* < 0.05) between the 96-well viable cell counts, the 48-well viable cell counts and the 24-well viable cell counts, despite plating the same number of cells (10 or 50 cells per well) in each experiment, for each cell line tested. The results are consistent and show that plating the same number of cells in a smaller volume results in greater outgrowth or clone formation in mAb4-treated cells, although both wells are still reduced in viable cell number compared to isotype-matched control mAb. Thus, while mAb4 still suppresses cell growth up to 500-1,000 cells per well, its effect is lessened when cell density is increased (Figure 2I). These data also show significant differences for each comparator case of same cell number per well *vs*. well size and volume. Control wells treated with isotype-matched mAb, plated at 1-5 cells per well, consistently produced clones with these cell lines. This experiment was expanded to include different mAb dilution experiments examining concentrations from 1 μg/ml to 20 μg/ml. The inhibition of growth of the mIgM-expressing B-cell line, CRL 1648, by mAb4 over ten days was tested at cell dilutions of 20, 100, 250, 500, and 1,000 cells/well. As shown in Figure 2I, mAb4 inhibited growth in the mIgM-expressing B-cell line, CRL 1648, for a 10-day period, but not when the concentration of cells plated was greater than 500-1,000 cells/well. These anti-clonogenic effects were confirmed by timed microscopic observations, which demonstrated that it was the inability of single cells to replicate (self renewal) and establish clones over the first 4-10 days of culture that was primarily responsible for reduced cell numbers. As a secondary effect, these mAb4 non-clonogenic cells subsequently became caspase-positive, undergoing apoptosis by days 8-10.

Similar experiments using control mIgG-expressing cells did not show any of the mAb4-induced biologic effects, and anti-PD mAb2, commercial polyclonal rabbit, mouse, or goat anti-huIgM was not anti-clonogenic with mIgM+ or mIgG+ cells in this assay, despite previous evidence that it internalizes the receptor (Figure 2B). These results suggest that the amino acid sequence determining functional growth inhibition is located in a neo-epitope defined by mAb4.

These MTT assays were extended to the cell lines shown in Figure 2, where a variety of mIgM B-cell lines were examined for mAb4-induced inhibition of cell growth and apoptosis. These experiments indicate that mAb4-induced inhibition was a general phenomenon in mIgM-expressing cells. mAb4 induced growth inhibition of mIgM-expressing B-cell lines, including CRL 1648 (Figure 2A), CRL 2958 (Figure 2D), CRL 1596 (Figure 2E), CRL 1432 (Figure 2F), CRL 2260 (Figure 2G), and CRL 1647 (Figure 2H).

**Table 5:** mAb4-Mediated Growth Inhibition in Limiting Dilution Assays Assessed by Well Size

Cell Lines	10 cells/ 0.3 cm^2^	10 cells/ 0.7 cm^2^	10 cells/ 2.0 cm^2^	50 cells/ 0.3 cm^2^	50 cells/ 0.7 cm^2^	50 cells/ 2.0 cm^2^
CRL 1648	**32**	**21**	**<1**	**67**	**25**	**<1**
CRL 1647	**41**	**18**	**<1**	**57**	**28**	**<1**
CRL 1596	**36**	**23**	**<1**	**66**	**18**	**<1**

Area sizes correspond to 96, 48 and 24 well plates, respectively.

## DISCUSSION

For B-cell leukemia and lymphoma, an abundance of evidence supports the contention that the BCRC itself drives the malignant phenotype [13, 32–37]. The concept that either an antigen encounter or persistent receptor stimulation is the basis for the constitutively activated BCRC state is supported by the finding that CLL cell immunoglobulin heavy chain variable region (IgVH) genes are often derived from a restricted repertoire [12,38–44]. As naïve B-cells encounter antigens in germinal centers, and the IgVH mutation-based maturation process is initiated, two distinct early B-cell subtypes emerge as progenitors of CLL. In one case, the malignant B-cell does not enter the somatic mutation phase [38,43]. Un-mutated IgVH cells have phenotypes consistent with weak poly-auto-reactive targets, including a subset that is self-reactive to the BCRC [38,43, 44]. In an environment of continuous stimulation of the BCRC and maximum BCRC-dependent signaling, patients are noted to have poor outcomes compared to the progenitor B-cells undergoing somatic IgVH mutations [44, 45].

In addition to these BCRC events, there is clear evidence that environmental factors play a critical role in providing different levels of co-antigenic stimulation, as well as other growth promoting factors [46–52]. Those cells undergoing somatic mutation may also exhibit auto-reactivity and respond to microenvironment-derived factors promoting growth in the absence of appropriate apoptosis [46–52]. For example, bone marrow and lymph nodes appear to be favorable sites for CLL proliferation while the blood appears to be less favorable [47, 53, 54]. The more recent observations of a drug-induced lymphocytosis linked to clinical response may correlate with increased vulnerability of these cells due to loss of bone marrow/lymph node environmental pro-survival factors [46–54]. Investigations into environmental regulatory factors and the constitutively activated BCRC state were the basis of the screening and testing of mAb anti-tumor effects at limiting dilution conditions as a model to tease out the individual factors that play a role in cell survival [28, 29,30]. Results presented here directly implicate external factors as determinants in cell survival in mAb4 treated cells (Table 5). This model facilitates further investigation and identification of the factors opposing mAb4-induced anti-tumor effects.

The identification of the BCRC as the initiating oncogenic stimulus is strikingly similar to the biology of Chronic Myelogenous Leukemia (CML), which is a model of an initial “one translocation event” mutation generating the single protein driver of malignancy [55]. Rational drug design targeting the translocation-induced constitutively activated TK in CML has lead to a marked improvement in clinical outcomes [55]. This example emphasizes the advantages of identifying the molecular structure initiating and maintaining the malignant phenotype. The continued growth-promoting stimulation by the activated BCRC would be expected to select for an accumulation of mutation variant clones in the downstream TK pathways, despite initial good clinical response to downstream TKI [16–18]. These finding suggest that anti-BCRC therapy may have maximum clinical benefit if administered early in the course of disease to suppress BCRC downstream stimulation, cell division, and mutational events, and thus control the disease in the chronic phase as has been demonstrated in CML therapy [55]. Early therapeutic intervention may, in general, be critical to achieve more robust outcomes. mAbs presented here provide an opportunity for direct targeting of the BCRC, the initiating factor in almost all B-cell malignancies.

“Chronic Active BCRC Signaling” has been identified in the activated B-cell-like (ABC) subtype of DLBCL. The BCRC was found to be present in micro-clusters on the cell membrane, driving cell growth [24–27]. The current SEM investigations reveal similar but smaller complexes of mIgM clusters present to variable degrees in all the B-cell lines tested in our panel (Figure 1G). This finding may suggest a more universal role for small mIgM clusters (“small micro-clusters”) as a mechanism for signal amplification and as a potential marker of prognosis [26]. As the SEM experiments represent results of glutaraldehyde-fixed cells, the clustered formations were not induced by mAb, but rather detected by our mAb panel. The mIgM cluster shown in Figure 1G may not be identical to those described for the ABC subtype of DLBCL, but may be functionally active. SEM investigations of mAb binding to DLBCL (not shown) demonstrated more intense gold clustering which may represent the classical micro-cluster, but further investigation is needed to classify these entities by size and function. As mAb4 induced growth inhibition, even in B-cell lines demonstrating increased clustering, this molecular event does not appear to confer resistance to mAb4 (Figure 2) nor does the clustering itself block the mAb4-binding epitope (Figure 1A-1C).

The depiction of the “mIgM-signalasome” has often been simplified, and betrays the complexity and redundancy that is known to be wired into B-cell activation, especially pathways from the stromal micro-environment [46–54], pathways across the membrane surface [56,57] and by interactions with numerous cytoplasmic mediators [4, 6, 58–62]. Taken together, investigations presented here, regarding the robust and specific biologic effects mediated by mAb4, demonstrate that the BCRC remains the central controlling element of oncogenesis (Figure 2). Cell membrane-associated proteins contribute to BCRC-dependent downstream signaling. For example, CD19 is a B-cell trans-membrane molecule that associates with the BCRC and is critical for B-cell activation [57]. Additionally, BCRC signaling is modulated by a net balance of CD19 and CD22 effects [63]. While CD19 is thought to amplify the signal generated through the BCRC, CD22 acts as an inhibitor [64–71]. These examples demonstrate how multiple extracellular and trans-membrane signaling pathways fine-tune the BCRC control of B-cell kinetics and the malignant phenotype.

A therapeutic strategy of BCRC cross-linking and internalization might be expected to disrupt or eliminate BCRC-dependent signal transduction. However, experimental evidence suggests that this is not the case and internalized BCRC continues to regulate cell processes [15]. Data presented here confirm these reports and demonstrate that the epitope directing cross-linking is critical in determining post receptor internalization events (Figure 2A *vs*. 2B). Anti-PD mAbs induce a state where cell surface mIg is no longer detected (by SEM using an anti-huIgM reagent). However, in this BCRC cell surface expression-negative state, no growth-inhibition effects are measured. While in contrast, the mAb4-induced BCRC negative phenotype results in growth inhibition and apoptosis (Figure 1I, Figure 2). Similarly, CD79α/β is critical in mIgM-directed signal transduction and is co-internalized with mIgM by anti-PD mAbs but continues to signal in its internalized state [15]. Anti-CD79 Abs have also been evaluated for internalizing capability, and growth-inhibitory effects. One anti-CD79α/β mAb, being developed as a “carrier mAb” to deliver cytotoxics to lymphoma cells, inhibits growth of B-cells [72]. In addition to the IgVH mutation prognostic status association, strong negative prognoses correlate to cytogenetic findings such as del 17p, associated with p53-functional impairment. Patients with tumors exhibiting p53 abnormalities have rapid disease growth and poor survival [73]. While this abnormality is believed to be remote from the BCRC and its immediate pathways, it is now well established that inhibition of Bruton's TK in the clinic reverses the rapid growth of these tumors, mediates response, and reduces the poor prognosis implications [73]. These findings further confirm the dominant role of inhibition of BCRC activation over distal regulatory cellular processes and survival outcomes. In the absence of growth stimulation induced by the BCRC, p53 anti-apoptotic abnormalities become less critical.

As progress has been made in selecting molecular targets for therapy, the therapeutic modalities themselves have evolved and have become more potent. New anti-CD20 Abs show increased efficiency of effector mechanisms of cytotoxicity. Engineered T-cell directed therapies, such as bi-specific [74] or T-cell chimeric antigen receptors [75], have demonstrated robust and specific targeted-restricted cytotoxicity, resulting in an almost surgical-precision resection of antigen-expressing tissue. However, the discovery of more tumor-specific targets has lagged in comparison to the increased potency of the cytotoxic mechanism, resulting in prospects of vast numbers of patients suffering with severe or complete B-cell depletion. The mAbs generated here offer a better focused targeting system more applicable to these potent drug or T-cell directed therapeutic modalities [74, 75]. Extensive specificity studies were conducted, which demonstrated the lack of epitope expression in epithelial or other lymphatic tissues and lack of cross-reactivities of these mAbs to unrelated proteins (Table 1A–1E). Data regarding the specificity of mIgM receptor targeting and internalization kinetics suggest that all four mAbs could reduce normal tissue toxicity when compared to currently utilized “mAb targeting agents”.

Here we show the internalizing kinetics of the mAbs, the identity of the mAbs reactivity with anti-huIgM sera and the completeness of mIgM internalization by each mAb (Table 2, 3, Figure 1A *vs*. 1I). Results of the SEM, protein G-RBCs, and cell adsorption timed experiments demonstrate that increasing times of mAb4 exposure (30 minutes at 37°C) specifically reduced the amount of cell surface mIgM to undetectable levels. Thus, with these mAbs, salvaging IgG- and IgA-expressing B-cells may be feasible, which would reduce the immune-suppression common with current treatments (Figure 2A *vs*. 2C).

In addition, an understanding of the spatial binding characteristics and epitope definition of each of the mAbs is critical in determining structural-functional relationships of their defined epitopes in macromolecules, and whether any of these epitopes have natural regulatory-ligands. The identification of biologically active epitopes may help detect and characterize possible natural binding regulatory ligands by affinity chromatography. For clinical utility, determining whether multiple mAbs can bind simultaneously to the target protein and what biological effects each may induce, will contribute to rational drug development. For example, if three mAbs from our panel each bind to different epitopes, and do not interfere quantitatively with each others' binding, then the amount of drug-bound-mAb delivered to the cell could increase substantially by using combinations of mAbs or even combinations of synergistic drug conjugates. Thus, further clinical studies will reveal the utility of the mAb panel presented here in targeted leukemia and lymphoma therapies.

## MATERIALS AND METHODS

### Generation of hybridomas and reagents

Immunogens and constructs: To isolate mAbs reactive with the target peptides, PDm-peptide EGEVSADEEGFEN, PDg-peptide ELQLEESCAEAQDGELDG, and immunogens carrying these peptides were constructed via glutathione-S-transferase methodology (GST) [76] (Promega, Madison, WI, USA; Rockland Immunochemicals, Limerick, PA, USA), or purchased as Multiple Antigen Peptide (MAP) PDm or PDg (Bio-Synthesis, Lewisville, TX, USA) and Keyhole Limpet Hemocyanin (KLH) PDm or PDg (Bio-Synthesis). GST, MAP, and KLH constructs were used as immunogenic covalently bound carrier proteins and sets of mice (six mice per set) were immunized with one protein, or combinations of the proteins carrying the target peptide. Due to relative hydrophobicity of the PDm peptide, both n-terminal and c-terminal KLH peptides were generated (Bio-Synthesis) for immunizations and for screening hybridoma supernatants. Eight constructs, including free EGEVSADEEGFEN (PDm), free ELQLEESCAEAQDGELDG (PDg), GST-EGEVSADEEGFEN, MAP-EGEVSADEEGFEN, KLH-EGEVSADEEGFEN, EGEVSADEEGFEN-KLH, KLH-EGENSADEEGFEN (mIgM isomer 1), and KLH-EGEVSADEEGFEN (mIgM isomer 2), were available for immunizations and for screening clones. The common overlapping sequences of PDe and PDd were also tested in screenings to further establish specificity (Bio-Synthesis). These multiple constructs, and the available free GST, MAP, and KLH, allowed for rapid screening and elimination of hybridoma clones reactive with carrier proteins or peptide conformations dependent on carriers or their linkers.

Immunization and hybridoma screening: Groups of mice, six mice per group, were immunized comprising 20 distinct immunization strategies of adjuvants, immunogens, and murine genetic backgrounds. Clones of interest were labeled according to fusion number, clone number, and immunization strategy.

Pre- and post-mouse-immunization serum was collected, diluted 1:100, and tested for peptide-specific activity in ELISA (Promega) to select mice for fusions [77]. Initial low titer sera and low peptide immunogenicity was observed and this finding was consistent with failed previous efforts to produce high affinity anti-PDm and anti-PDg mAbs. In contrast, this genetic background is appropriate to produce anti-mIgE mAbs [21, 78, 79]. Testing hybridoma supernatants also confirmed that when mAb was purified and adjusted for concentration, the mAbs were weak binders in ELISA assays. Screening assays were then designed to exclude low affinity mAbs. Selected mAbs were purified from hybridoma supernatants, utilizing rapid micro-protein A chromatography purification (Thermo-Fisher Scientific, Pittsburgh, PA, USA), and tested in protein concentration-dependent dilution ELISA assays [77]. New strategies, including extending the peptides into the μC4 domain 4 region (“extended peptide” 18mer (Bio-Synthesis)) to possibly capture conformational epitopes, using highly purified target protein for boosts, and testing a variety of mouse strains and new adjuvants, resulted in six mice with post immunization serum (1:100) titers of >1:10,000. These mice were selected for hybridoma generation and three clones were isolated that were active in binding to the eight target constructs, maintaining good ELISA reactivity in dilutional assays (< 10 ng/ml). Two clones were specific for mIgG and PDg.

Purified mIgM enrichment for immunization and screening: Upon generation and isolation of our first anti-PD mAb (mAb1) and completion of initial specificity testing, mAb1 was incorporated into immune-affinity chromatography (AminoLink™ Plus Immobilization Kit, 2 ml; Thermo-Fisher Scientific; Pierce Inc., Rockford, IL, USA) to enrich cell lysates for native mIgM. Patient Ficoll-Paque Plus (GE Healthcare Life Sciences, Piscataway, NJ, USA) purified CLL cells or cell lines (CRL 1648) were used as a source for native mIgM in 0.05% NP-40 (Sigma-Aldrich, St. Louis, MO, USA), and Perfect-FOCUS^TM^ - membrane protein extraction technology (G Biosciences, St. Louis, MO, USA; Pierce Inc.) cell extract fractions were used for assays and further mouse immunizations. Perfect-FOCUS^TM^ - membrane protein extraction technology removed ionic detergents, charged molecules, and cytoplasmic proteins by precipitating membrane proteins, which were subsequently collected by centrifugation (G Biosciences). The protein pellet was solubilized in immunogen buffer or pH 8.6 Tris 0.5N NaCl buffer for further mIgM purification by immune chromatography or applications as native mIgM for immunization. In these later immunizations, sets of mice were initially immunized with PD immunogens, KLH or MAP, and then boosted with lysate fractions containing mIgM to capture conformational epitopes.

The mIgM/mIgG molecules were proven to be expressed by the selected lymphoma cell line panel using RNA primers specific for PDm and PDg by RT-PCR, and were shown to be present in the cell membrane fraction by Western blot analysis [22]. PDd was tested by RT-PCR in select cell lines used for immunization and was found to be expressed in all lines tested. PDd, PDa, and PDg, and the common overlapping sequence of PDe, were generated and tested for mAb reactivity because purified CLL cell lysate fractions likely contained these PD constructs as contaminants (eg PDd). The mIgM and mIgG fractions were collected by Perfect-FOCUS™ - membrane protein extraction technology (G Biosciences), which yielded enriched mIgM or mIgG preparations from human CLL cells and cell lines CRL 1432, Namalwa mIgM-L Burkitt's lymphoma, CRL 1596, Ramos cIgM mIgM-L Burkitt's lymphoma, CRL 1647, ST 486, cIgM mIgM-K Burkitt's lymphoma, CRL 1648, CA 46, mIgM-K Burkitt's lymphoma, CRL 1649, MC 116, mIgM-L undifferentiated lymphoma, CRL 2260-HT, mIgM-K Diffuse mixed B-cell lymphoma, CRL 2958, SU-DHL-5, mIgM-L Diffuse large cell lymphoma, CRL 2632, Pfeiffer, IgG-K Diffuse large cell lymphoma, and CRL 2289, DB, mIgG-L Large B-cell lymphoma (Large B-cell lymphoma expressing mIgG), adequate for robust signals in ELISA studies. These assays were used to further demonstrate specificity and consistency of mAb reactivity to antigen, and lack of cross-reactivity.

Specificity screening: Using the above screening methodology, candidate mAbs were collected for further analysis. Comparative binding of purified mAb1, mAb2, and mAb3 demonstrated the highest specific ELISA signal of the anti-PDm mAbs when tested against each of the eight constructs in ELISA assays and the lysates from the mIgM-expressing cells listed in the previous section. Clones with lower anti-PDm reactivity in the ELISA assays were then tested against the purified Perfect-Focus^TM^ Immune Affinity Chromatography mIgM fraction (mAb1). One mAb, designated mAb4, showed enhanced reactivity with the purified fraction compared to the anti-PDm ELISA assay. To confirm mIgM specificity, these clones were tested against viable B-cell lines in HA [34], in SEM against +/− glutaraldehyde-fixed B-cells, and against purified sIgM (Promega) in ELISA assays.

### Candidate mAb panel

The Ab designated mAb1-1, produced by a hybridoma cell line from fusion 117, Ab designated mAb2-2b, produced by a hybridoma cell line from fusion 118, Ab designated mAb3-2b, produced by a hybridoma cell line from fusion 118, and Ab designated mAb4-2b, produced by a hybridoma cell line from fusion 119, were collected as the clinical candidate “panel of mAbs”. mAb1-1 was designated Group 1 as it was derived from a fusion comprising a mIgM-PD peptide-MAP immunogen only. All other Abs were designated as Group 2 as they were generated using boosts of various purified mIgM fractions in addition to the PDm-MAP immunogen. Further, these mAbs (mAb2, mAb3 and mAb4) were designated as Group 2b, as the purified mIgM was derived from cell line b (CRL 1648) extract.

### Cell line panels

Lymphoid cell panel: CRL 1432, Namalwa mIgM-L Burkitt's lymphoma, CRL 1596, Ramos, cIgM mIgM-L Burkitt's lymphoma, CRL 1647, ST 486, cIgM mIgM-K Burkitt's lymphoma, CRL 1648, CA 46, mIgM-K Burkitt's lymphoma, CRL 1649, MC 116, mIgM-L undifferentiated lymphoma, CRL 2260-HT, mIgM-K Diffuse mixed B-cell lymphoma, CRL 2958, SU-DHL-5, mIgM-L Diffuse large cell lymphoma, CRL 2632, Pfeiffer, IgG-K Diffuse large cell lymphoma, CRL 2289, DB, mIgG-L Large B-cell Lymphoma, and CRL 2568, H2.8 murine IgG1-K Myeloma were obtained from the American Type Culture Collection (ATCC, Manassas, VA, USA) specifically for these experiments. These cell lines were maintained in RPMI 1640 with 50 units penicillin/streptomycin, 2 mM glutamine, and 7.5-15% fetal bovine serum, and were incubated at 37°C in 5% CO_2_. The ATCC authenticates and tests cell lines provided for research as per their protocols.

Epithelial/non-lymphoid cell panel: Human colon cancer cell lines (six lines), human breast cancer cell lines (six lines), human lung cancer cell lines (six lines), human Melanoma cell lines (four lines), and human sarcoma cell lines (two lines) were obtained from our cell and tissue bank. The authenticity of these cell lines were tested by immune-phenotyping against a panel of defined mAbs.

Breast: BT 474, SK BR7, CaMa-1, BT-20, MCF-7, SK Br-3, MDA-MB 453, MDA- MB 436, and MDA-MB 468.

Lung: H64, SW1271, DMS 78, SK-LU-9, NCI H596, A549, NCI H1105, NCI H69, and DMS 53. Melanoma: SK MEL- 29 and MeWo.

Colon: T84, SW1222, Colo 205, Lim 1215, HT-29, DLD-1, SW1116, SW 620, SW 480, LoVo, HCT-15, and HCT-116.

Sarcoma: HT-1080 and U2OS.

Human serum samples from patients and normal volunteers (found specimens) were collected under Institutional Review Board-approved research and collected into our cell sera and tissue bank over many years.

### Ensuring the specificity of commercial regents

As these ELISA assays depend on the ability to detect a mouse Ig binding to a human mIgM, highly specific reagents are required. These assays comprise a set of target Ig proteins that are expressed in low concentrations and the detecting reagents are immunologically closely related Ig molecules of various species, thus cross-reactivity was a major obstacle and assay development was a critical aspect of specificity assessment. The ELISA assays were designed to enhance sensitivity for detection of mIgM by using a goat anti-huIgM capture antiserum bound to the solid phase. This methodology promotes the binding of both human cytoplasmic IgM (cIgM) and mIgM, which is present in low concentrations in NP-40 cell lysates. Increasing the total IgM binding capacity to solid phase resulted in enhanced assay sensitivity by increasing both cIgM and mIgM binding to plates (*p* < 0.05). To reduce non-specific background, the goat anti-huIgM capture serum needed to be pre-adsorbed with sepharose bound-mouse Ig.

The detection system consisted of a goat anti-mouse-Ig-HRP labeled reagent, pre-adsorbed with sepharose-bound huIgM and determined in separate assays not to be reactive with human mIgM or sIgM. However, the adsorbed goat anti-mouse Ig-HRP did detect mouse IgM mAb in hybridoma supernatant. When a mouse hybridoma IgM was present, it was found bound non-specifically to the solid phase as the pre-adsorbed goat anti-huIgM capture anti-serum also detected and bound mouse hybridoma IgM, rendering all IgM-producing mouse hybridoma clones as false positives in the screen. Thus, rather than continue with further adsorptions of reagents, an additional ELISA assay using protein G-HRP detection reagent was also tested as a control, as hybridoma IgM, sIgM, cIgM, and mIgM were not detected in this protein G-HRP assay. While the protein G-HRP assays removed positive and false positive IgM-secreting hybridoma results, the more sensitive goat anti-mouse Ig reagent allowed better detection of all mouse Ig subclasses. However, the protein G-HRP reagent required the capture reagent to be a F(ab)2 product to avoid protein G binding to capture reagent.

### Hemadsorption/Hemagglutinin assays

HA assays were carried out as described previously [77, 80]. As the HA assay was originally designed to rapidly assess Ab cell-surface binding to adherent target cells, the assay was adapted here for use in the generally non-adherent B-cell panel. Non-adherent or weakly-adherent test cells are first exposed to mAb in eppendorf tubes, washed, then exposed to protein G-RBCs at 4°C and examined under phase contrast microscopy on diluted feathered glass slides. HA resulted in large complexes of Ab-coated target cells cross-linked by protein G-RBCs. HA was scored as the number of un-reacted target cells (non-RBC rosetting cells) per un-reacted control assay target cells. This assay rapidly assesses if >99% of target cells are rosetted (hemeagglutination), and thus evaluates, on a single-cell basis, heterogeneity of mIgM expression. The protein G-RBC also weakly detected IgG-expressing B-cells without the addition of mAb, indicating its binding to surface-expressed mIgG. However, this binding was blocked by pre-incubation of protein G-RBCs with control IgG2a (mAbA33), which specifically blocked rosetting HA using protein G-RBCs, demonstrating the specific reactivity of the protein G-RBCs for cell surface Ig. Percent rosetted target cells = (control mAb tested cell un-rosetted - positive mAb tested cell un-rosetted) / control mAb tested cell un-rosetted X 100%. HA assays were scored using phase contrast microscopy (100x) as neg (< 1%), + (>1%), ++ (>90%), or +++ (>99%).

### ELISA sandwich assay of cell lysates

Of note is that all lymphoma cell lines used contain cytoplasmic IgM (variously glycosylated, but similar to serum IgM). Thus, these assays based on cell extracts neither confirm nor assess mAb specificity and are unable to distinguish mAb-specific reactivity between mIgM and serum or cytoplasmic IgM. Thus, cell membrane IgM binding specificity was determined by cell surface binding experiments utilizing viable cells. Protein labeling kits for HRP (Sigma-Aldrich; Pierce Chemicals) were used to directly label purified mAb for use in a solid phase ELISA assay, as per manufacturer's directions.

To test mAb/supernatant reactivity to extracts of CRL 1648-mIgM and CRL 2289-mIgG, a specific “murine Ig-adsorbed” goat F(ab')2 anti-human IgMFc or anti-IgGFc anti-sera capture Ab was attached to solid phase plastic in 0.5 M Tris 0.15 M NaCl pH 8.0, Nunc MaxiSorp^®^ flat-bottom 96 well plate (eBioscience, San Diego, CA, USA). The NP-40 lysate of CRL 1648 or CRL 2289, or the control breast cancer cell lysate, BT-474, was added to the wells. The CRL 1648 lysate provided human mIgM, and CRL 2289 lysate provided mIgG to bind the capture Ab. The wells were then washed three times. As BT-474 breast cancer cell line lysate did not provide protein binding to capture Ab, this was used as a control to test for cross-reaction of the mAb to the capture system, or cross-reaction of the capture system to the mAb detection reagents used in the ELISA assay [81, 82]. Due to excess cytoplasmic IgM or IgG contaminating NP-40 extracts of CRL 1648 or CRL 2289 respectively, additional membrane protein concentrating strategies, such as Perfect-FOCUS^TM^ were employed. Application of the Perfect-FOCUS^TM^ technology Membrane Proteins kit (G-Biosciences) after NP-40 lysis also reduced the NP-40 concentration in the enriched antigen fraction. For the majority of assays, CRL 1648 lysis fraction was used unless indicated otherwise (Table 1). Once anti-PD mAbs were established, immune-affinity column chromatography (mAb1) provided further enriched quantities of mIgM from Perfect-FOCUS^TM^ fractions (still partially complexed with CD79α/β, as determined by Western blot analysis), which was appropriate for *in vitro* assays and immunizations.

The Perfect-FOCUS^TM^ technology Membrane Proteins kit (G-Biosciences) was used as directed by the manufacturer. Cells (2 × 10^6^) were suspended in FOCUS™ Extraction Buffer-V (urea CHAPS buffer) and then lysed by sonication. Protein samples were treated with UPPA-reagents, a proprietary precipitation agent. Protein pellets were collected by centrifugation in a microcentrifuge tube and interfering agents were pipetted away with washes in Orgosol buffer provided with the kit. Precipitated protein was dissolved in 1 ml Extraction Buffer-V as a stock solution.

As above, to test the relative reactivity of the panel of mAbs to extracts of CRL 1648-mIgM and CRL 2289-mIgG, a specific murine Ig-adsorbed goat anti-human IgMFc or anti-IgGFc anti-sera capture Ab was attached to solid phase plastic, in 0.5 M Tris 0.15N NaCl pH 8.0 (eBioscience). The NP-40 lysate/Perfect-FOCUS^TM^ fraction of CRL 1648 or CRL 2289, control human serum lysate, or control breast cancer cell lysate, BT-474, was added to the wells. The CRL 1648 lysate provided human mIgM, and CRL 2289 provided mIgG, to bind the capture Ab. The wells were then washed three times. Purified mAb was then added to wells in which specific mAbs had bound to the captured human mIgM or mIgG, which was in turn bound by either the capture anti-human IgMFc or IgGFc (Promega) forming a goat anti-human-IgMFc-mIgM-mAb complex or goat anti-human-IgGFc-mIgG-mAb complex. The mouse mAb was then detected with specific goat anti-mouse Ig, labeled with HRP (preabsorbed with human IgM-sepharose or human IgG-sepharose). Hybridoma supernatants were added, and specific mAbs detecting captured human mIgM or mIgG, which was bound by the capture anti-human IgMFc or IgGFc, formed a goat anti-human-IgMFc-mIgM-mAb complex or goat anti-human-IgGFc-mIgG-mAb complex. The mouse mAb is then detected with specific HRP labeled goat anti-mouse Ig (preabsorbed with human IgM-sephrose or human IgG-sepharose) (Thermo Fisher Scientific). Other positive cell extracts from our B-cell panel yielded similar results.

Specificity of reactivity was further confirmed using mIgE derived from human B-cell line SK007 (human B-cell line expressing mIgE without mIgM) by NP-40 lysis of SK007 cells, and was tested with ELISA using specific goat anti-human IgE capture Ab. To eliminate the possibility of reactivity with transmembrane or cytoplasmic domains (KVK), HA assays were carried out, and fluorescent microscopy (FM), using fluorescent labeled goat anti-mouse Ig pre-absorbed with CLL cells, was used to detect mAb bound to fresh viable CLL cells. Additional assays were carried out with selected hybridomas to assess relative affinity by testing mAb purified from supernatant added to wells at 0.1 μg/mL and serially diluted 2X. This allowed for removal of clones secreting low affinity mAbs. Inhibitory ELISA assays were carried out by incubation of purified mAb with inhibitor for 30 mins at 4°C and centrifuged to remove complexes prior to addition of mAb to ELISA plates. mAb concentration was adjusted based on dilutional effects of the inhibitor to maintain a concentration of mAb at 0.1 μg/ml.

### mAb-induced internalization experiments

Using a sensitive alternative methodology, such as adsorption, to assess relative residual cell surface mIgM levels at various time points after mAb exposure, viable B-cell lines were exposed to either glutaraldehyde (cells fixed as per SEM protocol) or PBS (Table 4). In glutaraldehyde-fixed cells, no internalization occurs and cells exposed to mAb4 first do not allow subsequent mAb4-labeled HRP adsorption as the epitope is already blocked by mAb4. Thus, testing the adsorbed solution of mAb4-HRP in ELISA assays after absorption by mAb4-coated antigen of glutaraldehyde fixed cells, yields maximum binding, which is then set as 100% binding for each cell line (Table 4, Row 1). Groups of viable (PBS) cells were exposed to 10 μg/ml mAb4 at 4°C for 5 mins (Table 4, Rows 3 and 4), or at 37°C for 5 mins (Table 4, Rows 5 and 6), 15 mins (Table 4, Rows 7 and 8), or 30 mins (Table 4, Rows 9 and 10). At the end of the incubation periods, cells were washed with cold PBS pH 7.0 (Table 3, Rows 5, 7, and 9) or 0.5 M acetate 0.5 N NaCl pH 4.0 solutions (Table 4, Rows 6, 8, and 10) for 10 mins on ice, then washed in pH 7.0 PBS to re-equilibrate cell pellets to physiologic conditions and fixed with glutaraldehyde and washed again prior to adsorption [83].

### Western blot analysis

Western blot analysis was carried out as previously described [22]. Samples were boiled in electrophoresis sample buffer containing 0.0625 M Tris-HCl (pH 6.8), 10% glycerol, 2% SDS, and 5% BME, for 10 mins and then separated on 12% SDS-PAGE gels (mini-protean; Bio-Rad, Richmond, CA, USA) at 100 V until the dye front reached the bottom, then transferred overnight to nitrocellulose membranes (47 mA) (Bio-Rad). Membranes were blocked with a 5% w/v solution of non-fat dry milk in TPBS for 2 h at room temperature. The blots were then probed with primary Ab according to the titers provided by the manufacturer. The blots were washed once with TPBS and a 1:10,000 dilution of secondary Ab (either goat anti-rabbit alkaline phosphatase or goat anti-mouse alkaline Med Oncophosphatase) (Sigma-Aldrich) was then added. The blots were developed in the alkaline phosphate substrate, NBT/BCIP (Promega). Ponceau S (Sigma-Aldrich) blot staining and Coomassie blue (Sigma-Aldrich) gel staining after transfer allowed for an assessment of efficiency of the transfer. 0.1 M TRIS, 1% NP-40 substitute (Sigma-Aldrich), 0.01% SDS, 1 μg/mL Aprotinin (Roche Applied Science, Indianapolis, IN, USA), and 0.1 μM PMSF (phenylmethanesulfonylfluoride; Sigma-Aldrich) was also tested to resolve membrane proteins. Standard RIPA buffer with Triton X-100, and sodium-deoxycholate conditions and non-denaturing conditions were each used to separate hydrophobic membrane proteins. Boiling in phenol was also tested to separate membrane proteins.

### Cell growth/mAb internalization assays

Plates were tested every two days, wells were pooled for MTT analysis, and relative viable cell number was determined by MTT assay of each experiment (Promega; Life Technologies, Grand Island, NY, USA) [22]. Relative MTT optical density was plotted for each time point for the 12 pooled cell values for each experiment [22]. EnzCheck Caspase-3 assay kit (Life Technologies) was used to detect apoptosis as per manufacturer's directions and pooled well samples were assessed to increase detection. mAb internalization assays were carried out as previously described with modifications [83] and cells were examined by mAb absorption assays and parallel SEM assays.

### SEM

The SEM studies were prepared to parallel binding studies, such as the HA, and to investigate internalization of cell surface mAb. For these studies, fixation with glutarahdehyde or modified Karno*vs*ky's fixative, 2% paraformaldehye/2% glutaraldehyde in 0.1 M phosphate buffer was used to block biologic processes such as internalization instead of using reduced temperature. AURION Ultra Small Immunogold (AURION Biosciences Inc., Yonkers, NY, USA) was used for detection of cell surface mAb. Carbon-coated, poly-l-lysine-coated glass 13 mm round cover-slips were introduced into 6-well plates containing the test cell samples. Cells were incubated with mAb both before and after cell fixation with glutarahdehyde, which was followed with a serum-free media rinse. The serum-free media was then pipetted off. 2.5% glutaraldehyde in 0.1 M cacodylate buffer was then added, followed by PBS (1 mM phosphate buffer, 150 mM NaCl) 0.1-0.2% AURION BSA-c™ 15 mM NaN_3_ pH 7.4. Prior to scanning, ultra small gold (anti-mouse IgGFc) was added for 3-4 hours and then silver enhanced with an SE-EM kit and SE-LM kit as per manufacturer's directions (AURION Biosciences Inc.). Images were viewed on a Zeiss Supra 40 field emission SEM microscope.

### Statistical considerations

Student's t-test was used to assess statistical validity of ELISA data points. All data points were calculated from values across 12 wells in each of three experiments performed.

HA/ELISA cell lysates assays (Table 1): A separate statistical test is carried out for each mAb compared to each control shown: isotype control, anti-mIgG mAb11.1, and anti-huIgM. Values that are bolded are statistically significant (*p* < 0.05).

Molecular constructs-immunogens: Statistical significance is measured for molecular construct-immonogens relative to their respective control for each target tested, including PD (of m-mIgM or g-mIgG), mIgM, KLH, MAP, Perfect-Focus^TM^ (Perfect FOCUS^TM^ cell extract), and IA.

Biological specimens ELISA: Statistical tests were performed comparing the mAbs to polyclonal anti-huIgM reagent (Table 1, last column).

Inhibition of direct mAb binding to Perfect-Focus^TM^ assay: Statistical significance was calculated using comparisons of mIgM-PD, KLH-mIgM-PD, and Perfect-Focus + IA, each in a test of mAb-HRP reactivity pre-blocked by unlabeled excess mAb (e.g., mAb1-HRP was blocked by unlabeled mAb1 and similar results were found for each HRP-labeled mAb by its partner mAb). Serum tested included Waldenstrom's Macroglobulenemia serum (W-Ms) that contained 4.2 g/dL IgM, CLL serum that contained 22 mg/dL of IgM, and DLBCL Indolent non-Hodgkin's lymphoma (iNHL).
